# Responsible Oversight of Human Stem Cell Research: The California Institute for Regenerative Medicine's Medical and Ethical Standards

**DOI:** 10.1371/journal.pmed.0040114

**Published:** 2007-05-08

**Authors:** Geoffrey P Lomax, Zach W Hall, Bernard Lo

## Abstract

California voters recently approved $3 billion over 10 years for public funding of stem cell research through the California Institute for Regenerative Medicine (CIRM). Geoffrey Lomax and colleagues discuss the principles that guided the CIRM regulations.

Restrictions on United States federal funding have thrust state governments into a leadership role in funding and establishing standards for human embryonic stem cell (hESC) research. In 2004, California voters approved $3 billion over 10 years for public funding of stem cell research through the California Institute for Regenerative Medicine (CIRM), making CIRM one of the world's largest supporters of this pioneering new field. CIRM recently adopted strong, comprehensive, and legally binding regulations for the human stem cell (hSC) research that it funds. Box 1 describes the historical background of CIRM regulations. Because other states and jurisdictions may also be developing standards for hESC research, consideration of the principles that guided the CIRM efforts and the innovative measures that it enacted may be useful to others.

## Overall Objectives

Several overall objectives, in addition to the goal of setting high ethical standards, guided formulation of the CIRM regulations.

**Encourage research institutions and researchers to develop best practices for ethical conduct of hSC research.** Research oversight is decentralized in the United States, in that Institutional Review Boards (IRBs) at each research institution take primary responsibility for reviewing all proposed projects involving human participants. hESC research, however, raises ethical issues that are beyond the mission or expertise of IRBs. Among these, for example, are the scientific justification for using oocytes and embryos to derive new hESC lines and concerns that transplantation of hSCs into the brains of animals might result in transfer of human characteristics. CIRM requires that a Stem Cell Research Oversight Committee (SCRO) be constituted at each institution with appropriate expertise to review, approve, and oversee CIRM-funded stem cell research. Institutions have considerable flexibility in coordinating the work of the SCRO and IRB.

The regulations recognize that hSC research is a rapidly advancing field and that there is little experience overseeing such research. To encourage SCRO committees to develop and adopt best practices, the CIRM regulations set performance standards rather than prescriptive standards. For example, the regulations require that those who donate oocytes for hESC research not only give consent, but show comprehension of what they have agreed to. The regulations do not specify how the assessment of comprehension is to be accomplished—this is left to researchers, with the approval of IRBs. The expectation is that with experience and evaluation, best practices for donor consent will be developed and broadly applied.

**Avoid unnecessary regulatory burdens.** US oversight of research with human participants has been criticized for requiring excessive documentation and procedures that add time and expense but do little to protect against ethical lapses. The CIRM regulations try to avoid unnecessary regulatory burdens. To avoid duplicative review, some specified human stem cell lines are acceptable for CIRM-funded research without further review: stem cell lines approved by the US National Institutes of Health, which were derived before August 2001, as well as cell lines approved by the United Kingdom Human Fertilisation and Embryology Authority, the UK Stem Cell Bank, or those meeting the Canadian Institutes of Health Research Guidelines for Human Pluripotent Stem Cell Research.

Some types of human stem cell research—such as research with adult and cord blood stem cells and in vitro research with embryonic stem cells—do not raise novel ethical concerns and therefore do not require in-depth review by the SCRO. However, in-depth SCRO review is required for research that raises complex ethical issues [1–6], including research involving oocytes and embryos, the derivation of new pluripotent stem cell lines, and the introduction of human stem cells into humans and nonhuman animals.

Documentation is also tailored to the degree of ethical concern. A research institution must maintain a record of every donated egg or embryo and any products of somatic cell nuclear transfer that have been produced or used in CIRM-funded research. Such strict accounting helps prevent misappropriation of oocytes or the illegal use of somatic cell nuclear transfer for reproductive purposes. While burdensome reporting is not required, CIRM retains the power to conduct investigations and to penalize violations.

**Involve the public in developing regulations.** Given the public's role in providing the research funding and the level of interest in hESC research, public involvement in the formulation of the regulations was important. CIRM found various ways to engage the public during the process of drafting and revising the regulations. First, all SWG meetings were public meetings, at which the public had access to all written materials, such as briefing papers and draft guidelines, and were invited to comment on each topic discussed. These discussions often involved vigorous give-and-take discussions involving both working group members and members of the public. Second, CIRM held a series of meetings around the state at which the public was invited to offer input and comment on ethical issues related to stem cell research. The most frequent comments concerned the ethical use of human oocytes for nuclear transfer research. Finally, after the regulations were drafted, there was a 45-day period of formal public comment, during which CIRM responded in writing to all suggestions made. Many suggestions were incorporated into the final regulations. The extensive exchange with the public resulted in a stronger document that reflected both professional and public input.

**Be consistent with existing laws, regulations, and ethical guidelines.** The Working Group was very conscious of existing regulations and sought to harmonize the CIRM regulations with them, so that researchers and their institutions would not face contradictory requirements from different funding sources or regulatory bodies.

**Box 1. Background of the CIRM Regulations**

In November 2004, the California Stem Cell Research and Cures Act (Proposition 71) was passed by voters. The Act established the California Institute for Regenerative Medicine and authorized US$3 billion to support human stem cell research. The Act created the Standards Working Group (SWG) to recommend scientific, medical and ethical regulations to govern research funded by CIRM. In July 2005, the SWG recommended adoption of the April 2005 National Academies of Science Guidelines for Human Embryonic Stem Cell Research as interim regulations. The SWG held eight public meetings between July 2005 and July 2006 to develop permanent regulations. In addition to receiving public comment during meetings, the SWG obtained public comment on each proposed revision of the interim regulations; these changes were posted on the CIRM Web site. During this time CIRM provided 78 formal responses to over 125 written comments. In addition, the final regulations were subject to review and approval by the California Office of Administrative Law. The final regulations took effect in November 2006.

**Facilitate collaboration to accelerate scientific progress.** To facilitate research cooperation, CIRM wanted to uphold international standards but also recognize and respect differences in detail. This issue arose most acutely in defining the requirements for hSC lines used by CIRM-funded researchers but derived with other funding. CIRM did not insist on exact conformity to CIRM standards, but defined core requirements for hSC lines that CIRM-funded researchers might use [6–8]. For example, imported hESC lines need not meet CIRM's heightened consent requirements for oocyte donors. The question of whether oocyte donors should be paid is one issue on which there is extensive disagreement. In this case, the ballot measure authorizing the Institute, as well as other California laws, prohibit payment to donors of research oocytes beyond reimbursement for expenses.

## Innovative Features of the CIRM Regulations

**Informed and voluntary consent.** Because human embryonic stem cell research is controversial, prospective donors need to be informed as completely as possible about possible research uses of embryos, gametes, and tissue that they might donate. If donors have stated restrictions on the future uses of donated materials, CIRM-funded researchers must respect these. Because it is difficult to foresee all future uses, however, researchers are free to utilize only materials whose donors have consented to all future research uses that are approved by scientific and ethical review bodies. This provision strikes a balance between respecting the informed preferences of donors and maximizing the scientific benefit from research funding.

**Protection of oocyte donors.** Donation of oocytes to derive new stem cell lines raises heightened ethical concerns about medical risk and the voluntary and informed nature of consent [6,9]. For example, in California, some legislators and members of the public have charged that infertility clinics downplay the risks of oocyte donation. Ethical concerns were amplified by the 2005 Hwang scandal, which featured a high incidence of complications from oocyte retrieval, lack of informed consent, undue influence, and payments to oocyte donors, as well as egregious research misconduct [9,10].

In addition to obtaining consent, researchers must ascertain that oocyte donors *comprehend* eight essential features of the research. In other research settings, research participants often fail to understand the information in detailed consent forms [11–13]. CIRM thus reasoned that disclosure, while necessary, is not sufficient to guarantee informed consent. Evaluating comprehension is feasible, as it has been carried out in other research contexts, such as in HIV prevention trials in the developing world [1,14]. According to testimony presented to CIRM, evaluation of comprehension has also been carried out with respect to oocyte donation for research.

The regulations strive to minimize conflicts of interest for physicians carrying out oocyte retrieval. The physician performing oocyte retrieval may not be the principal investigator or have a financial interest in the outcome of research. A different conflict of interest occurs if a donor provides oocytes simultaneously both for CIRM-funded research and for infertility treatment (either for herself or another woman). In this situation, the optimal reproductive success of the woman in infertility treatment shall not be knowingly compromised. Fertility treatment thus takes unequivocal priority over research.

CIRM regulations require that research institutions assure free treatment to oocyte donors for direct and proximate medical complications of oocyte retrieval, which include hyperovulation syndrome, bleeding, infection, or complications of anesthesia. The US does not have universal health insurance. As a matter of fairness, women who undergo an invasive procedure for the benefit of science and who are not receiving payment should not bear any costs for the treatment of complications. Compensation for research injuries has been recommended by several US panels [12], but has not been adopted because of difficulties calculating long-term actuarial risk and assessing intervening factors that could contribute to or cause adverse events. However, requiring free care for complications of oocyte donation is feasible. Commercial insurance policies are available to cover short-term complications of oocyte retrieval [15]. CIRM funding may be used to pay for such insurance.

In summary, CIRM's regulations are designed to provide strict oversight without stifling research. The full provisions are available online (http://www.cirm.ca.gov/laws/default.asp). These regulations, which were developed with extensive public input, are a critical first step in increasing public trust and support for human stem cell research.

## 

**Figure:**
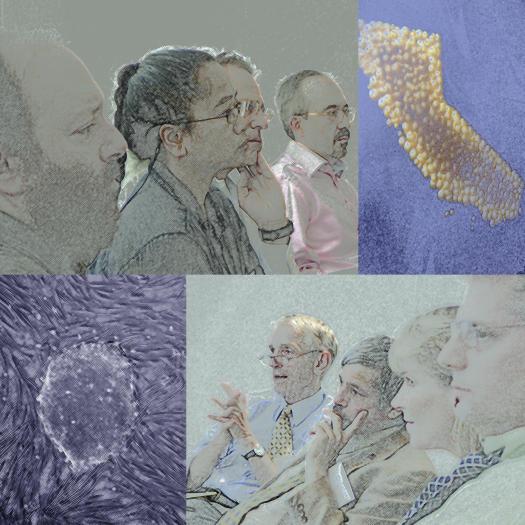
CIRM developed comprehensive medical and ethical standards for stem cell research through a process involving extensive expert and public consultation. (Credit: The Burnham Institute & CIRM)

## Abbreviations

CIRM: California Institute for Regenerative Medicine
hESC: human embryonic stem cell
hSC: human stem cell
IRB: Institutional Review Board
SCRO: Stem Cell Research Oversight Committee
SWG: Standards Working Group
